# Acellular fish skin grafts in the treatment of diabetic wounds: Advantages and clinical translation

**DOI:** 10.1111/1753-0407.13554

**Published:** 2024-04-25

**Authors:** Chenyu Zhao, Mengyi Feng, Martin Gluchman, Xianghe Ma, Jinhao Li, Hui Wang

**Affiliations:** ^1^ Department of Ion Channel Pharmacology, School of Pharmacy China Medical University Shenyang China; ^2^ Department of China Medical University‐The Queen's University of Belfast Joint College, School of Pharmacy China Medical University Shenyang China; ^3^ School of Pharmacy Queen's University Belfast Belfast UK; ^4^ School of Pharmaceutical Science Wenzhou Medical University Wenzhou China

**Keywords:** acellular fish skin, advanced bandage, diabetes, diabetic wounds, fish skin graft, wound healing

## Abstract

Diabetic wounds cannot undergo normal wound healing due to changes in the concentration of hyperglycemia in the body and soon evolve into chronic wounds causing amputation or even death of patients. Diabetic wounds directly affect the quality of patients and social medical management; thus researchers started to focus on skin transplantation technology. The acellular fish skin grafts (AFSGs) are derived from wild fish, which avoids the influence of human immune function and the spread of the virus through low‐cost decellularization. AFSGs contain a large amount of collagen and omega‐3 polyunsaturated fatty acids and they have an amazing effect on wound regeneration. However, after our search in major databases, we found that there were few research trials in this field, and only one was clinically approved. Therefore, we summarized the advantages of AFSGs and listed the problems faced in clinical use. The purpose of this paper is to enable researchers to better carry out original experiments at various stages.

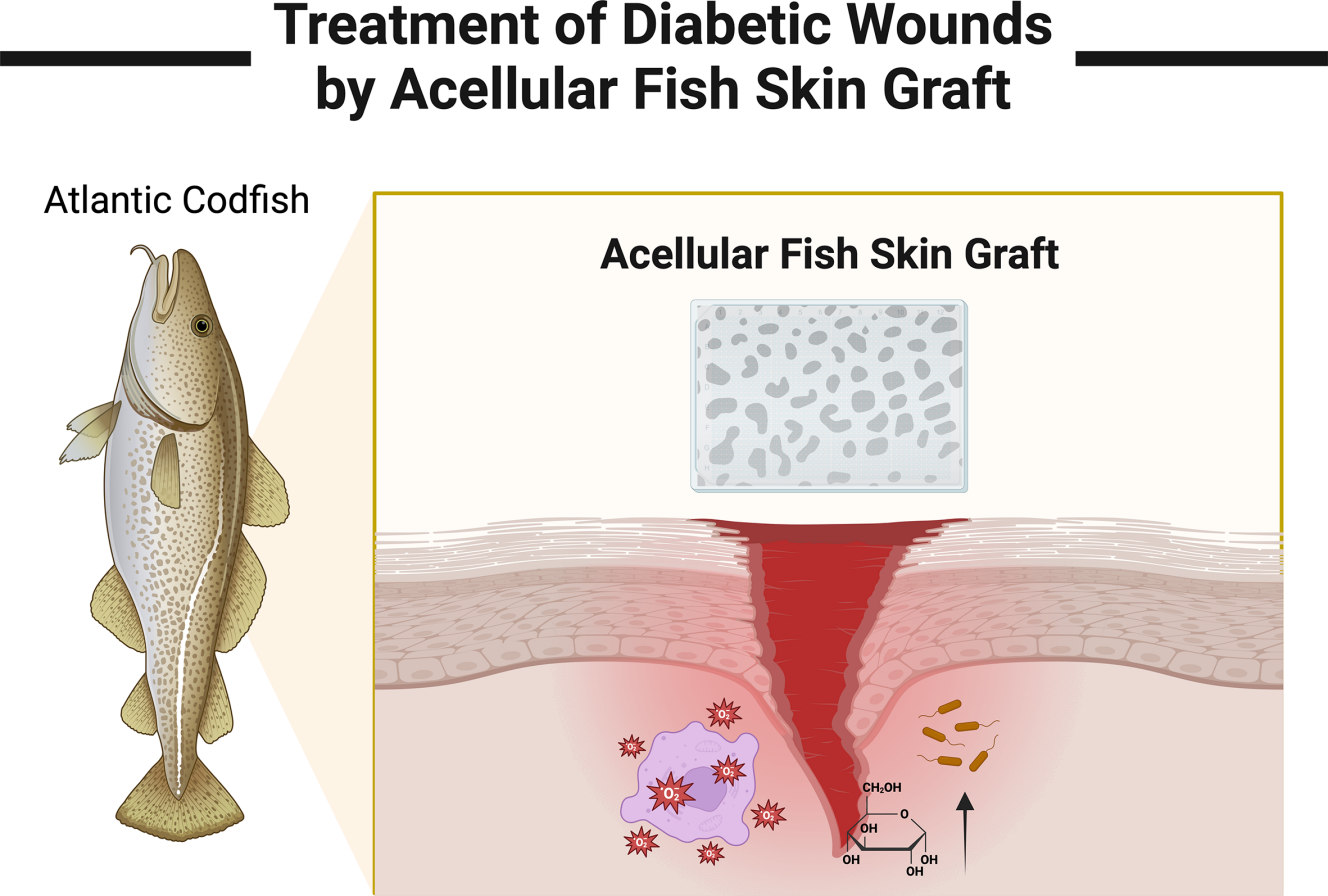

## Graphical abstract

Highlights

- Concisely included the latest research on the role of acellular fish skin grafts (AFSGs) in treating diabetic wounds, providing readers with a basic framework,
- This review outlined the therapeutic advantages and prospects of AFSGs,
- This work emphasized the role of cost‐effectiveness in the treatment of diabetic wound dressings.
- We summarized the shortcomings and corrective measures of current AFSGs clinical trials.
- We explored the resistance that may be encountered by AFSGs in various stages of clinical studies.

## 1 INTRODUCTION

In the past 3 decades, the global prevalence of diabetes has risen rapidly, and this trend is expected to reach 7.7% in 2030.^1^ China is one of the countries with the fastest increase in the prevalence of diabetes in the world. As one of its major complications, diabetic wounds greatly affect the quality of life of Chinese diabetic patients and the operation of local medical systems.^2^, ^3^ The largest lesion of diabetic wounds is the foot of the human body, and its 5‐year mortality rate is even comparable to that of some cancers.^4^ The healing process of normal wounds usually goes through the sequence of coagulation, inflammation, proliferation, and remodeling. However, due to the influence of high blood glucose concentration in diabetic patients, the wound will be in the stage of inflammation and tissue infection for a long time. The high blood glucose concentration of the body will lead to excessive glycosylation of proteins, promote the expression of proinflammatory factors, and cause oxidative damage to local tissues. In addition, it also inhibits the proliferation of fibroblasts, keratinocytes, and endothelial cells, making the wound unable to heal. Peripheral neuropathy, as another feature of diabetes, can lead to the loss of protective sensation in patients, especially in the foot. Inappropriate shoes or minor injuries may induce diabetic foot.^5^ The extensive and prolonged impact of pathological conditions on the acute wound will evolve into chronic wounds, eventually forcing doctors to take passive treatment such as amputation, which will cause great harm to the patient's body and mind.^6^, ^7^, ^8^ Therefore, researchers urgently expect to develop an efficient treatment to alleviate the pain and burden of patients.

**FIGURE 1:**
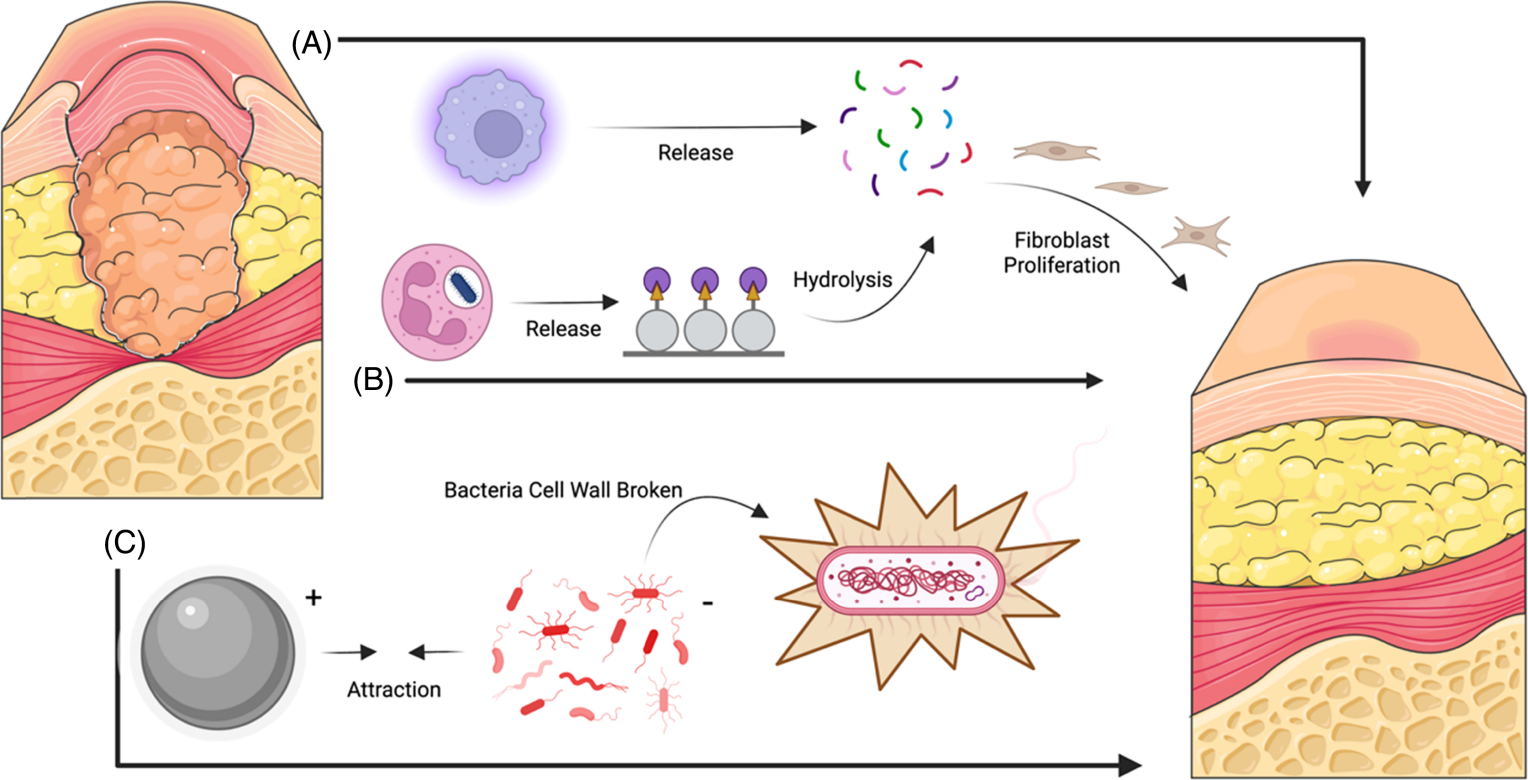
The wound repairing process of traditional dressings. Endogenous: (A) Macrophages performed phagocytosis and released small molecule peptides to help fibroblasts to form new collagen; (B) Neutrophils released protein hydrolases to enzymatically degrade antigens into small molecule peptides, which in turn promoted tissue regeneration. Exogenous: (C) Silver ions and bacteria can be adsorbed to each other due to charge difference, followed by cell wall peptidoglycan reaction, and eventually rupture, which led to bacterial death.

**FIGURE 2:**
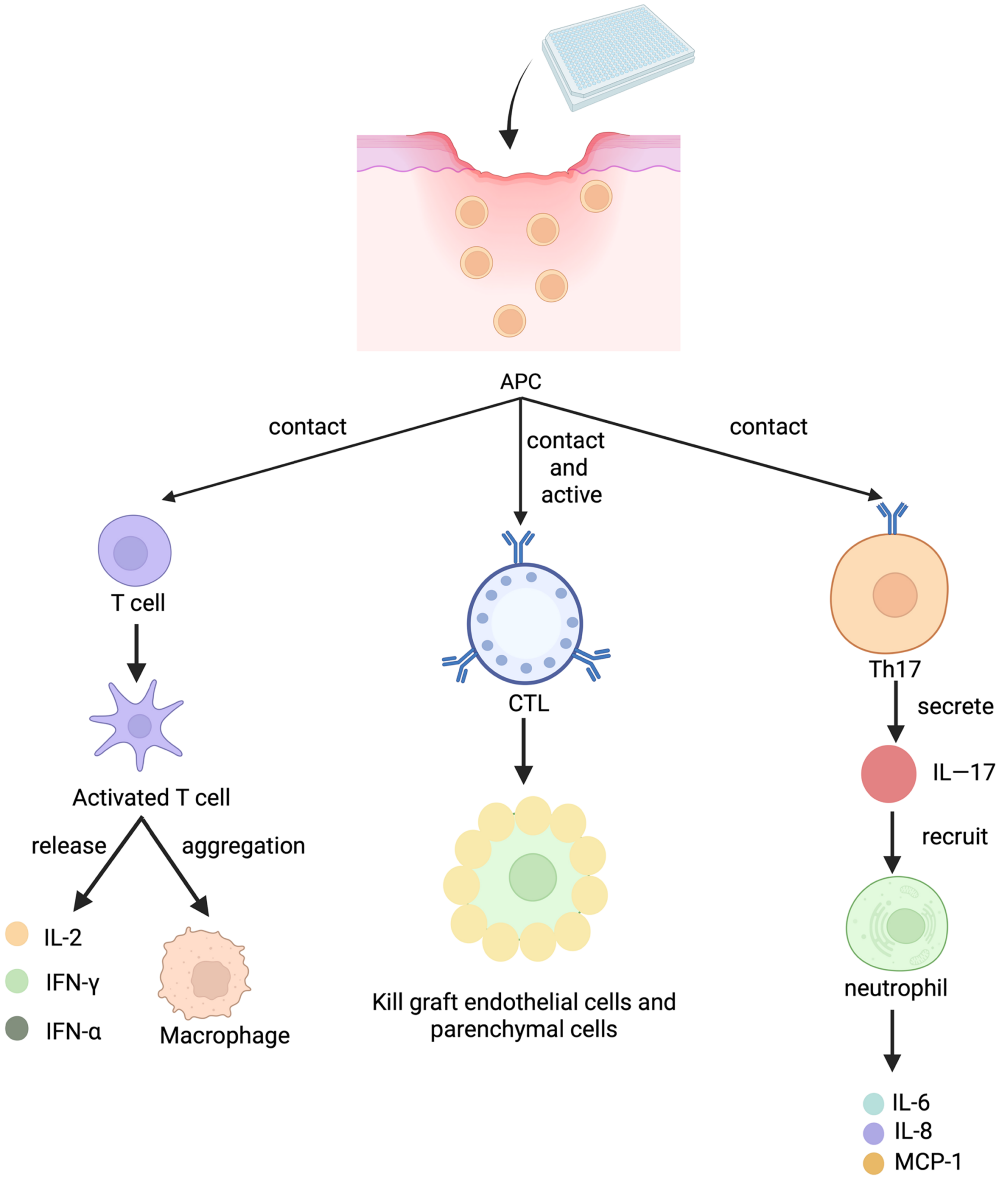
Mechanism of immune rejection induced by xenografts. APC, antigen‐presenting cell; CTL, cytotoxic T lymphocyte; IFN, interferon; IL, interleukin; MCP‐1, monocyte chemoattractant protein‐1.

Wound treatment can be adapted to different interventions according to the area and depth of the wound. In the past, collagen dressings and silver sulfadiazine cream commonly used in clinical practice need to be replaced frequently, which is likely to cause secondary damage to the wound surface of patients and cause some trouble to health care providers^9^ (Figure 1). Many studies have shown that skin transplantation has a significant improvement in the treatment of diabetic wounds.^10^, ^11^, ^12^ Skin transplantation can be divided into homotransplantation and xenotransplantation according to the source of substitutes. Autologous transplantation needs to create new wounds in different parts of the patient's own body. Primarily, this is not a priority for the treatment of large‐area wounds. Second, this attempt is obviously unlikely to be carried out in diabetic patients. Allogeneic transplantation will face the risk of graft rejection because the patient's own immune system is likely to identify the allogeneic skin graft as a foreign body and then produce a certain immune response. Xenografts can carry viruses on animals, leading to wound infection in the host^13^ (Figure 2). To encapsulate, these skin transplantation methods have a certain degree of side effects on the wound and cannot achieve the ideal healing of the wound.

KerecisOmega3 was approved by the Food and Drug Administration in 2013 for the management of various types of wounds and has been gradually extended worldwide in recent years. The dressing is based on the skin of North Atlantic cod, which is decellularized to remove the corresponding components that can stimulate the human immune system, and then an acellular fish skin graft (AFSG) is obtained. It contains many regeneration‐promoting substances such as collagen and fibrin, as well as the most significant omega‐3 polyunsaturated fatty acids, which are considered to play an important transitional role in the wound healing stage. At present, some studies have proved the essential role of AFSGs in wound management. However, only KerecisOmega3 is currently approved in the clinical stages, and it is no wonder what is troubling researchers in moving it into clinical use on a large scale. Researching the major databases, we found there is a lack of articles extensively discussing the challenges in the use of AFSGs in the treatment of diabetic wounds toward clinical application.

This review will systematically discuss the various resistances we may encounter in the clinical process of AFSGs. First, we conclude a comparative review of the advantages of AFSGs. Then, we divide the process of clinical trials into two parts: the design of grafts and the limitations of clinical trials. The difficulties that researchers will face at each step and some unavoidable systematic errors are considered in detail.

## 2 THERAPEUTIC ADVANTAGES OF AFSGs

**FIGURE 3:**
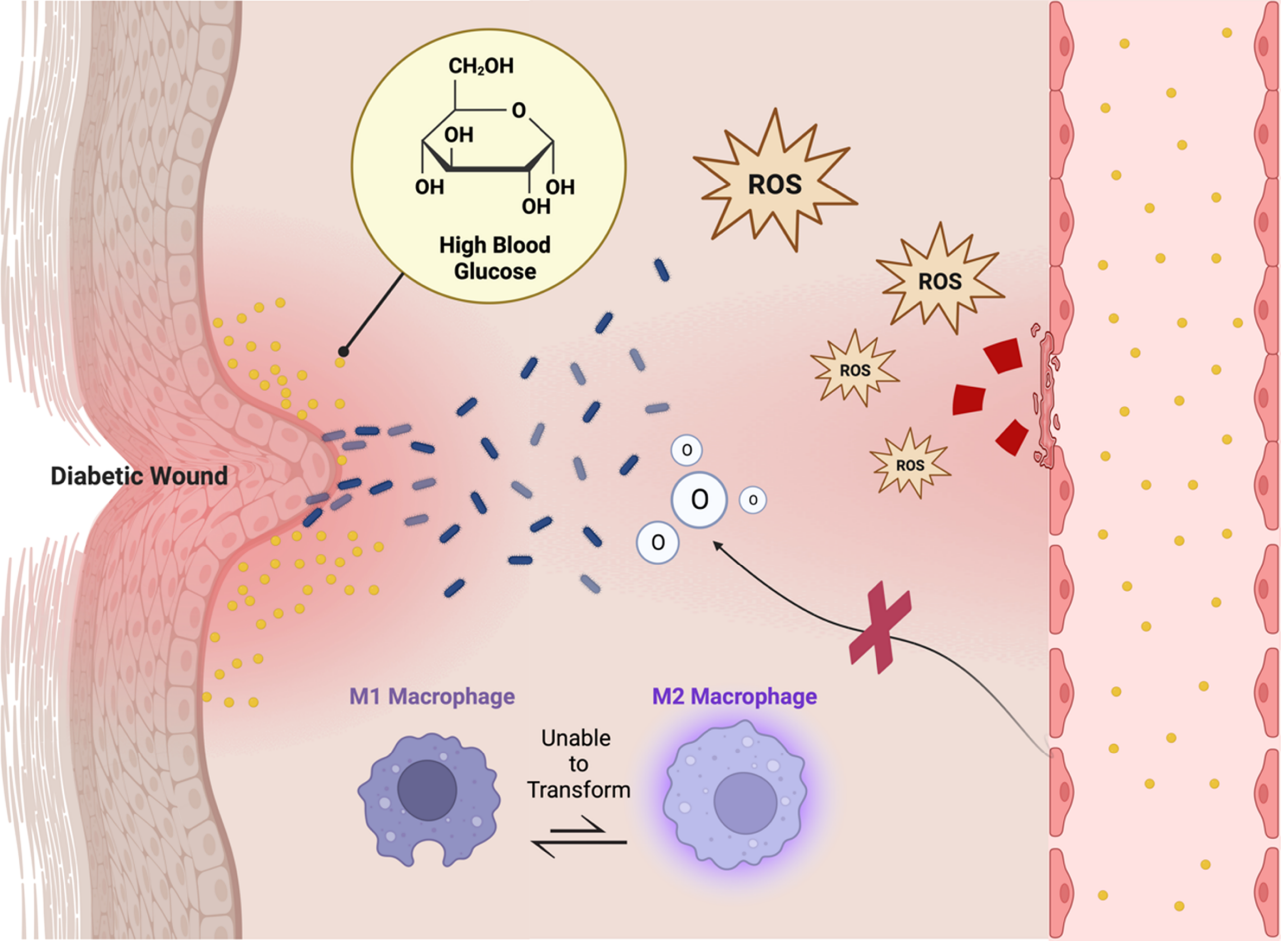
The pathological mechanism of a diabetic wound: When a diabetic wound becomes infected, the high concentration of blood glucose in the body promotes bacterial colonization and biofilm formation, leading to uncontrollable inflammation. In addition, biofilms are present in most ulcers, deteriorating the overall wound environment. Persistent inflammation due to reactive oxygen species (ROS) production is also an important cause. ROS are produced by damaged inflammatory cells. Sustained hyperglycaemia in diabetic wounds produces advanced glycation end products in the blood, which directly induces the overproduction of ROS. In addition to causing chronic inflammation, excessive ROS impair angiogenesis, promote cellular senescence, and impede reepithelialization. Vascular damage makes it difficult to transport oxygen and nutrients to the site of the wound. These wounds usually occur on the extremities, especially the feet.

Wound healing is a dynamic and complex biological process, which involves many cell types, extracellular components, and cytokines. Diabetes affects multiple physiological mechanisms of this process, so the entire wound is in microcirculation and macrocirculation disorders. The focus of research on the treatment of diabetic wounds should be on the implementation of understanding the pathological mechanism, so as to develop targeted interventions.^14^ Due to the significant weakening of the body's glucose metabolism, which cannot produce and transform many functional proteins, the loss of wound healing potential is the main effect of diabetes. The high blood glucose concentration in the body also promotes the colonization and proliferation of bacteria in local tissues, induces biofilm to cover the wound, and deteriorates the wound environment. Advanced glycation end products can directly catalyze the excessive production of excessive reactive oxygen species (ROS), followed by the imbalance of M1 macrophages that are difficult to convert into M2 macrophages. ROS can also directly damage the blood vessels of the wound, making nutrients and oxygen difficult to transport to the wound.^15^ The aforementioned pathological process of diabetic wounds is the root cause of the development of wounds into chronic wounds (Figure 3). If this is not solved, it is difficult to obtain all the effects of endogenous tissue regeneration factors and external intervention methods in the later stage.^5^ The management of general diabetic wounds is divided into active and passive treatment. The former is represented by insulin injection, negative pressure wound therapy and hyperbaric oxygen therapy, which are conventional treatment methods for lowering blood sugar and increasing oxygen content in wounds. The latter are wound dressings that focus more on anti‐infection and tissue healing in the later stages of wound management.^16^ A UK‐based population cohort study showed that the 5‐year mortality rate of diabetic wounds was as high as 42%, so the traditional treatment model was no longer advantageous, and it was difficult to see how this could be optimized.^17^ Thus, we need an unconventional and innovative treatment.

### 2.1 AFSGs can be used as a bridge between active and passive treatment

The most superior feature of AFSG is its lipid characteristics. The main components of many omega‐3 polyunsaturated fatty acids are docosahexaenoic acid (DHA) and eicosapentaenoic acid (EPA). These two substances have antibacterial and anti‐inflammatory properties and can even fight against methicillin‐resistant *Staphylococcus aureus*.^9^, ^18^ Implantable medical devices have brought revolutionary breakthrough to the development of medicine, but the risk of infection behind them is what we need to avoid. As a new type of implantable skin substitute, AFSG greatly avoids the spread of virus after decellularization. In addition, some recent studies have shown that omega‐3 polyunsaturated fatty acids also have certain antibacterial abilities. Coraça‐Huber et al tested the antibacterial toxicity of EPA and DHA to S.epidermidis in a study and found that high concentrations of unsaturated fatty acids have killing activity on planktonic cells and can cause inhibition of biofilms. They calculated that 5–0.625 mg/L EPA could completely kill hemolytic streptococcus, and the same concentration of DHA also showed stronger activity.^19^ Omega‐3 unsaturated fatty acids can reduce the gene expression of *Staphylococcus aureus*.^20^ At present, research on AFSG in the treatment of diabetic wounds has not explored its antibacterial role. Although most of the mentioned studies are limited to the anti‐infection test in artificial joint surgery, they are all based on the activity of omega‐3, so we can assume that AFSG can indeed play a more significant antibacterial effect. In addition to antibacterial property, Soleimani et al evaluated the response of patients with diabetic foot ulcers to linseed oil omega‐3 unsaturated fatty acid supplements in terms of anti‐inflammatory regulation of diabetic wounds. Compared with placebo (paraffin), omega‐3 unsaturated fatty acid supplements caused a significant decrease in the length, width, and depth of wound ulcers after a 12‐week intervention. As we expected, not limited to wound care, regular supplementation of omega‐3 unsaturated fatty acids will significantly increase plasma total antioxidant capacity and glutathione concentration, which closely fits the ideal blocking treatment for ROS overproduction.^21^ McDaniel et al also demonstrated that supplementation of omega‐3 fatty acids for up to 4 weeks in healthy people can lead to an increase in proinflammatory cytokine interleukin‐1 beta (IL‐1β), which can regulate fibroblast proliferation and accelerate collagen synthesis.^22^ Therefore, we can see that AFSG does have a significant effect on the active treatment of diabetic wounds.

**FIGURE 4:**
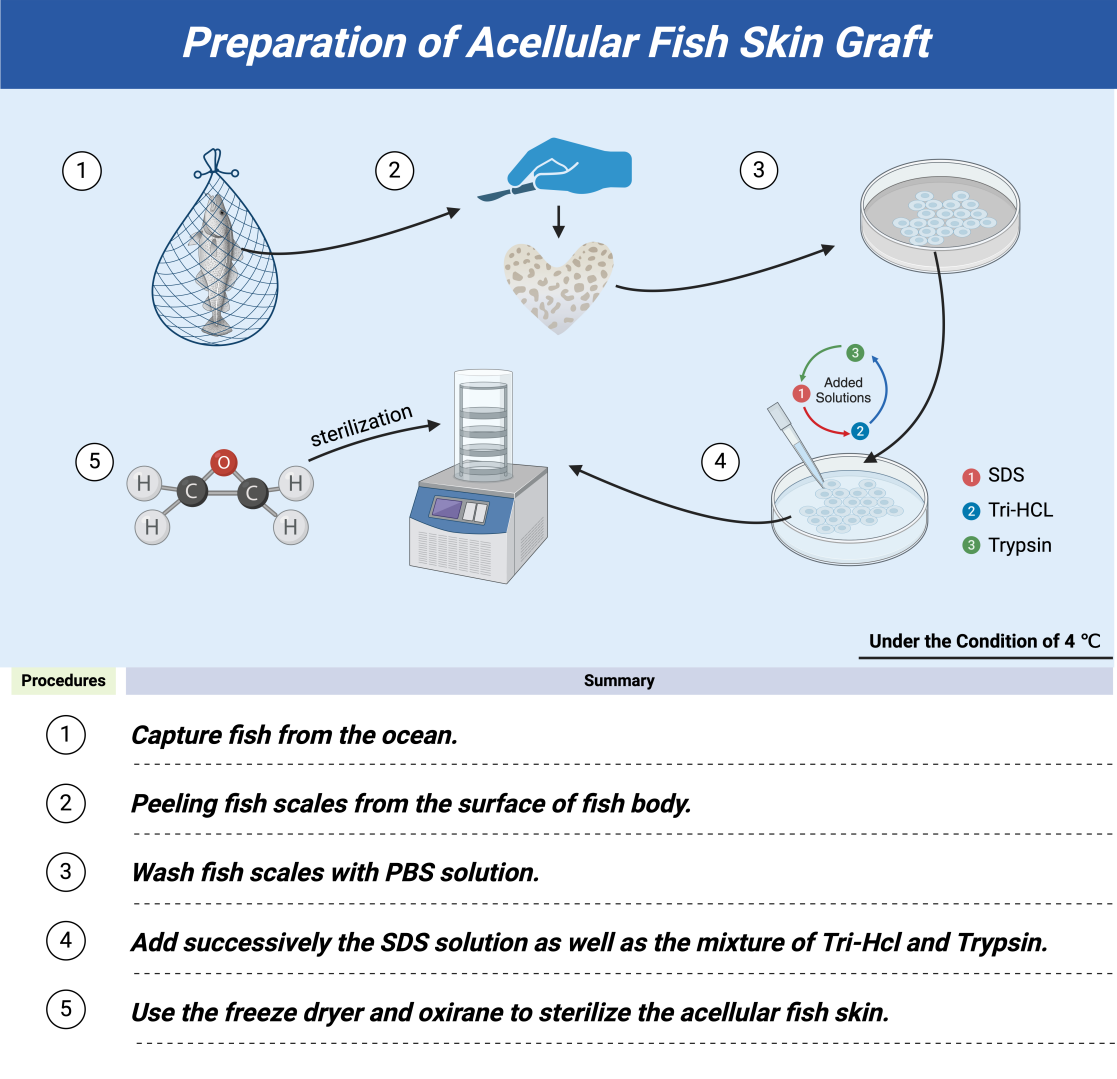
The preparation of acellular fish skin graft. Skin of fresh fish was cleaned and cut into 4 × 4 cm small pieces, placed in 4°C PBS and 1% antibiotic mixture and treated within 24 h. The skin was shaken in 2.5 U/mL dispase in PBS for 3 h to detach the epidermis. The skin was rinsed with deionized (DI) water and shaken in 1% sodium dodecyl sulfate (SDS) in PBS for 6 h to lyse the cells and release cellular contents. The skin was then gently scrapped to physically remove the epidermis. The skin was rinsed with DI water and shaken in 25 mL nuclease (or Tri‐HCL, trypsin) and residual contaminants. The skin was rinsed with DI water and lyophilized in a freeze‐dryer for 24 h. The lyophilized skin was further dried in a vacuum chamber for 1 h to remove any condensation. This final product was termed AFSG.^58^

The skin of Atlantic cod is very similar to that of humans, which has three layers: epidermis, middle layer, and basal epithelium. Each layer of tissue has noteworthy porous structure and an average thickness of 450 microns. Because no chemicals and detergents are used in the decellularization process, the Atlantic cod skin retains good mechanical properties^18^, ^23^ (Figure 4). Compared with other wound dressings, AFSGs are more suitable for the treatment of diabetic wounds at different depths. Christy et al proved that AFSGs can accelerate the closure of deep wounds, which can increase the early blood flow of wound healing. Compared with fetal bovine dermis, AFSGs mainly increase the hydration level of deep wounds.^24^ Ibrahim et al compared the efficacy of AFSGs with other wound healing techniques in a study. They found that AFSGs had better wound healing than alginate collagen dressings, 1% silver sulfadiazine cream, and other xenotransplantations.^13^ The biological activity of omega‐3 unsaturated fatty acids does not include the role of fibroblast proliferation at the wound, but it can increase the migration of host adipose stem cells. In the process of wound healing, the matrix of AFSGs plays a significant role.^18^ The main content of cod skin matrix is type I collagen, which not only has good thermal stability and can better adapt to the wound environment, but also has the effect of efficient combination with proinflammatory cytokines.^25^ Lullovede et al compared the therapeutic effects of standard care (collagen dressing) and AFSGs transplantation with the percentage of wound healing in the twelfth week as the end point of treatment. At the sixth week, the AFSGs transplantation group reduced the wound area by 72.8%, which was significantly better than the standard nursing effect (32%).^26^ Therefore, we can see that the AFSGs is also extraordinarily effective in wound care therapy. The AFSGs has a good effect in both active treatment and passive treatment. It breaks the barrier of traditional treatment methods and plays a remarkable transitional role in both aspects.

### 2.2 Cost‐effectiveness considerations of AFSGs

According to statistics, nearly 40% of health expenditure in developing countries and 12%–15% in developed countries is spent on diabetic patients. Therefore, we can see that the treatment of diabetes has caused a significant burden on the economy of each country.^27^ In the care of diabetic patients in the United Kingdom, about 8.8% of the expenditure is related to diabetic ulcers and amputations. Although there are no relevant data to explain the actual expenditure of diabetes wound care in China, combined with the aforementioned statistical trends, we can see that diabetic wounds in China will only reflect a more serious health financial burden.^28^, ^29^ A systematic review of reviews and research papers on diabetic foot ulcers reported in five developed countries (France, Spain, Italy, Germany, and the United Kingdom) found that amputations account for the highest proportion of expenditures and are the main source of the economic burden of diabetic wound management.^30^ After the diabetic wound develops into a chronic wound, the final measure basically taken is amputation, not only causing an economic burden for the family and society but also physical and mental damage to the patient, and the quality of life in the future is extensively reduced. Therefore, we need a good treatment to avoid the occurrence of chronic wounds and slow down the economic pressure of family and society.

When considering the cost‐effectiveness of AFSG transplantation, we should not only consider its production cost but also establish a new model to evaluate the wound healing rate, because the average wound area will gradually decrease with time, and the production cost of AFSGs will not change for a period. Only by accelerating the healing rate of diabetic wounds can the overall cost‐effectiveness be improved. Compared with the use of alginate collagen alone, the cure rate of diabetic foot ulcers treated with alginate collagen combined with AFSG can be tripled, which will save an average of $ 2818 per patient per year (the annualized cost of AFSGs per patient is $ 13 926; the annualized cost of collagen alginate therapy is $ 16 744). With the increase of the cure rate, the possibility of amputation is also reduced, so it can be seen that the AFSG has great potential for clinical application.^31^ In addition, other studies have used different models for discussion, and the overall cost‐effectiveness of AFSGs is considered to be better.^26^, ^32^ However, we summarized two problems: first, we need to find a mature wound dressing to be used with AFSG and establish the most reasonable and economical treatment guidelines. Second, the intervention treatment process of AFSG requires the mediation of professional nursing staff. This is an expenditure factor that is difficult to incorporate into the cost–benefit model. More research is still needed to optimize the existing statistical model. In conclusion, AFSGs are important interventions to avoid chronic wounds and reduce family and socioeconomic pressures and have good prospects.

## 3 CHALLENGES FACED BY AFSGs IN THE PRODUCT DESIGN STAGE

### 3.1 There are too few mature products on the market at present

A good dressing should have excellent biological activity and be cost effective for various deep wounds. However, KerecisOmega3 is the only product that has been approved for mature AFSGs on the market, which is thought provoking. What hinders the early research and development of researchers? North Atlantic cod skin is known to be rich in omega‐3 unsaturated fatty acids, which can reduce local inflammation and promote angiogenesis. EPA and DHA are involved in changing the main process of wound healing.^25^ McDaniel et al randomly assigned a total of 1.6 g of EPA and 1.2 g of DHA per day to patients aged 18–45 years. Five days later, significant vesicular epithelialization of the patients' wounds was observed, indicating that the improvement of wound healing.^22^ In addition, studies have shown that omega‐3 unsaturated fatty acids can not only accelerate the healing of ulcers but also prevent the recurrence of ulcers. Although many studies have proved that omega‐3 fatty acids in fish oil have a positive effect on the epithelialization of wounds, they may inhibit the deposition of collagen protein in the later stage. Therefore, the therapeutic effect of omega‐3 on wounds is still controversial.^33^, ^34^, ^35^ Alipoor et al investigated the effects of collagen‐rich beverages containing omega‐3 unsaturated fatty acids on wound healing, metabolic biomarkers, and adipokines. The results showed that continuous supplementation of collagen hydrolysates could improve wound healing and scar quality, but the omega‐3 experimental group had no actual effect on wound healing. Therefore, if there is only one mature product on the market, it will not produce market competition. First of all, it is not conducive to the technical update of the product. In addition, all clinical trials use this product, which is difficult to avoid the subjective bias of data caused by the company's funding. For example, Burger et al tested the effect of oral administration of EPA‐rich oil on skin wound healing in diabetic mice and proved that EPA supplemental oil caused a delay in wound healing by increasing IL‐10, so KerecisOmega3 still has room for optimization. The biological activity of omega‐3 unsaturated fatty acids is still in an exploratory stage. More experiments are still needed in this field to understand the role of omega‐3 unsaturated fatty acids, and even to refine the regulation of EPA and DHA in wound inflammation.^36^, ^37^

### 3.2 The original material of AFSGs is limited

The low cost and biocompatibility of aquatic collagen indicate the potential application of AFSGs in biomaterials.^38^ Presently, most of the research experiments are based on AFSGs derived from Atlantic cod, which is undeniably influenced by the mature product Kerecis®Omega3. The preparation of AFSG and the process of extracting fish bioactive peptides are cumbersome and expensive.^39^ In the stage of optimizing a listed product, more material selection will enable research and development personnel to have more choices and design ideas. In recent years, tilapia has often been proved to have the potential to become a good xenograft. Li et al extracted acid‐soluble collagen and other substances in the skin of tilapia and transplanted tilapia collagen ultrafine fiber matrix scaffolds under the dorsal skin of 96 mice. The implanted scaffold was completely degraded after 20 days without any pathological inflammatory tissue reaction. The main component of tilapia acellular products is type I collagen.^40^ Related experiments have also developed a few tissue engineering products based on tilapia collagen scaffolds.^41^, ^42^, ^43^ Kangning et al found that tilapia AFSGs have low immunogenicity and risk of viral infection compared with other biological materials. In the immunohistochemical results, tilapia AFSGs significantly promoted the expression of epidermal growth factor, fibroblast growth factor, nerve growth factor, and CD31 skin‐related healing factors and induced and promoted the proliferation of skin fibroblasts.^44^ In addition, 250 μg/mL silver nanoparticles is the best disinfection concentration for xenotransplantation.^45^ Some researchers have also studied grass carp to a certain extent, but unfortunately, no research‐based experiments on diabetic wounds have been carried out in tilapia or grass carp.

### 3.3 Can AFSGs be chemically modified and drug loaded?

Although AFSG retains its collagen and natural structure, these two characteristics create a suitable microenvironment for tissue repair and regeneration, but its wound healing effect still has room for improvement.

Growth factor‐rich plasma, as an endogenous substance, can well promote tissue regeneration and has been widely used in clinical practice.^46^, ^47^, ^48^ If the growth factor‐rich plasma is combined with AFSGs, it will better promote the healing of diabetic wounds. For example, Biazar et al made a corresponding attempt, and no allergic and inflammatory reactions occurred throughout the process. Growth factors accelerate wound healing by mobilizing marginal cells. Vascular regeneration can provide oxygen and nutrition to the wound, and it is completely closed at 28 days.^49^ In addition to bioactive components, the mechanical properties of dressings are also important, which play an crucial role in providing a three‐dimensional microenvironment for the former one to support cell activity. Due to the similarity of the structure and function of the extracellular matrix between fish and humans, the application of AFSGs is promising. Due to the similarity of extracellular matrix structure and function between fish and humans, the application of AFSG is prospective. However, the AFSGs will undergo different enzyme environments during the process of re‐decellularization and will undergo a certain degree of degradation after human administration, which greatly affects the mechanical properties of the AFSGs. Abbasnezhad et al reported an attempt to improve acellular fish skin grafts by using chemical crosslinking agent 1‐Ethyl‐3‐ (3‐dimethylaminopropyl) carbodiimide in different amounts. They found that with the increase of the concentration of crosslinking agent, the mechanical strength and degradation time of AFSGs were greatly improved, but too high concentration would cause certain cytotoxicity.^50^

There is still a long way to explore the comprehensive improvement of drug‐loading capacity and scaffold mechanical degree of AFSGs in the future. From the current research, the drug‐loading attempt of AFSG is feasible. For the treatment of a diabetic wound, an excellent dressing can not only promote wound healing but also achieve a reduction in local blood glucose concentration, from the fundamental treatment of disease.^51^ Therefore, many experiments are attempting to encapsulate different clinical first‐line drugs, and even try some theoretically mature small molecule drugs. The antidegradation ability of the fish skin scaffold affects the number of dressing changes to a certain extent, which in turn affects the cost‐effectiveness of the excipients. Excellent chemical modification can improve the drug loading capacity of the scaffold and affect the therapeutic effect of a dressing.

## 4 DISCUSSION ON THE SHORTCOMINGS OF AFSGs IN CLINICAL TRIALS

**FIGURE 5:**
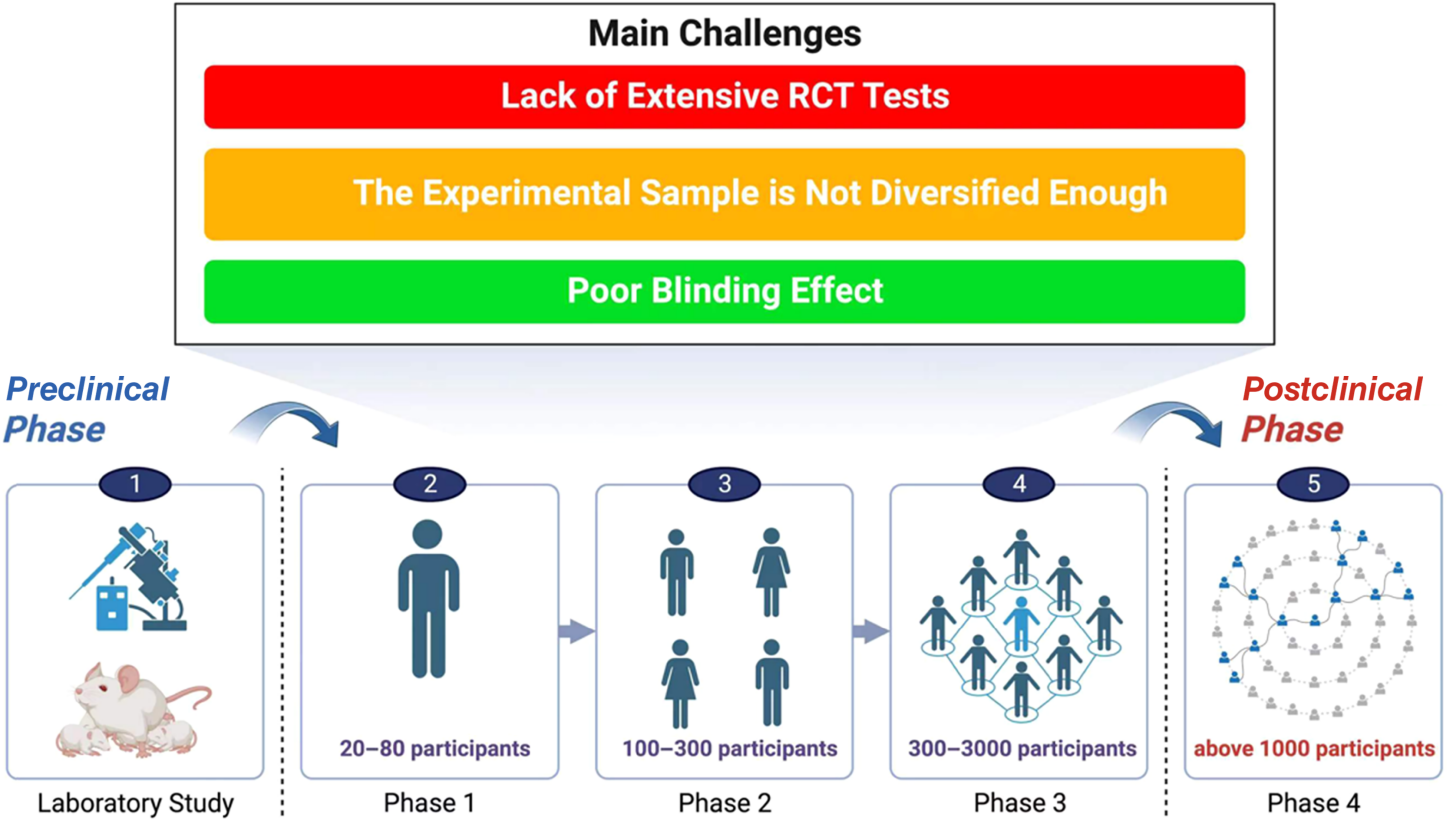
The main challenges faced by AFSGs during the clinical phase: (a) lack of extensive RCT tests; (b) the experimental sample is not diversified enough; and (c) poor blinding effect. RCT, randomized controlled trial.

AFSGs will encounter different degrees of random bias and systematic bias in clinical trials (Figure 5). The former is an unavoidable factor in clinical trials, whereas the latter is due to the influence of subjective factors of researchers and included patients, both of which are the main reasons affecting the objectivity of test data.^52^ At present, few clinical trials have been carried out. We summarize that the experiments proposed by the researchers in each trial are insufficient to carry out dialectical induction and provide a reference basis for the researchers who carry out subsequent trials.^53^

First of all, it is necessary to expand the scale of the trial, which is the most important for an excellent clinical trial data. At present, the maximum number of diabetic wound patients included in the trial is only 102 people. If there are not enough samples, the conclusions drawn and the deficiencies summarized in the trial are biased.^54^ Woodrow et al reported that the lack of control groups and the randomness of the experiment made them analyze only through the control groups of other similar studies. However, there are few similar studies in this field, and the criteria for inclusion of samples are different, which greatly affects the accuracy of the experiment. It is worth noting that they conducted simultaneous treatment interventions for patients with acute wounds and patients with chronic wounds and found that the early treatment intervention effect of acute wounds was extremely obvious, and the wound area of patients with chronic wounds was also reduced by 40% within 6 weeks. Therefore, we believe that in future clinical trials, the number of patients can be increased and divided into acute and chronic wound group to better explore the therapeutic effect of AFSGs on wounds in different periods.^16^ Lantis et al mentioned that future research needs to include more research sites, contain patients in various stages of diabetic wounds, and carry out long‐term follow‐up studies. All the current studies have occurred in the United States, Switzerland, and Iceland. There is no diversity in the population representation and location of the patients, and the proportion of Black and Hispanic population is obviously insufficient.^31^ If the study of large cohorts is rather difficult to conduct, can the result‐based model assist the experiment? Zehnder et al established a result‐based model that can predict which wounds can heal or not in advance based on changes in wound area after 4 weeks, in line with current guidelines for poor wound response, to improve prognosis and save costs for hospitals.^54^

Furthermore, it is difficult to cover the blinding during the administration of patients^55^ Due to its special treatment, patients in the double‐blind trial easily know that their intervention is the experimental group or the control group, which has a great impact on the trial. In the treatment of wound healing, the impact of the placebo effect is worth discussing. Mathur et al hypothesized that under the condition of no treatment in the control group, informing the patient in advance of the treatment received will enable the patient to have more complete reepithelialization after 7 days. The results showed that the patient's subjective influence had little effect on wound healing.^56^ Similar experiments have also shown that the placebo response has little effect on wound healing.^57^ Although these two trials have proved that the placebo effect has little effect on it, we still need to pay attention to the differences between diseases, because there are still too few clinical trials on diabetic wounds.

The clinical trials of AFSGs for the treatment of diabetic wounds need to be carried out in a large number in the future. The problems that can be summarized at this stage are still not breakthrough enough, but they can have certain relevance.

## 5 CONCLUSION AND PERSPECTIVE

After searching for a selection of databases, we systematically summarized the advantages of AFSGs in the treatment of diabetic wounds. Traditional diabetic wounds can achieve local tissue regeneration only after difficult treatments such as hypoglycemic, antibacterial, and inflammation regulation. AFSGs can achieve the overall convergence of treatment and are superior to traditional wound management methods. In addition, AFSGs belong to xenotransplantation, but the pretreatment of acellularization makes it avoid side effects such as virus and immune system disorder. Previous studies have shown that in the process of diabetic wound management, the quality of life of patients is not only the risk of amputation but also the economic burden of long‐term care.

Our review suggests that AFSGs have extremely high economic benefits and can also be combined with traditional treatment guidelines to create more efficient treatment interventions. However, only KerecisOmega3 is currently available on the market, indicating that there is a certain resistance in the design of preclinical and clinical trials of AFSGs, so we have carried out the corresponding induction. There are still some controversies in the basic research on the bioactive components of fish skin, which indicates that there is still room for improvement in the preparation of AFSGs. The existing products are all derived from Atlantic cod. If there are more ways to obtain fish skin, it will be beneficial to reduce costs. In addition, combined with the design experience of previous biological dressings, if a certain number of active drugs are loaded in the AFSGs, it may have a more positive wound healing effect. In terms of clinical trials, more randomized controlled trials are still needed to distinguish acute wounds from chronic wounds during the inclusion of patients, expand the inclusion samples while accurately including the conditions, and achieve the diversity of the population, which will be a big challenge.

AFSGs have excellent therapeutic effects in other types of wounds. We firmly believe that soon, researchers will overcome difficulties and achieve a combination of theory and practice to design a personalized treatment for diabetic wounds.

## AUTHOR CONTRIBUTIONS

Chenyu Zhao drafted and wrote the manuscript, Mengyi Feng designed the figures of the manuscript, Xianghe Ma and Jinhao Li provided the information of the manuscript, Martin Gluchman was responsible for language check. Hui Wang revised the manuscript. All authors read and approved the final manuscript.

## FUNDING INFORMATION

National Natural Science Foundation of China, Grant/Award Numbers: No. 81702611 to Hui Wang.

## CONFLICT OF INTEREST STATEMENT

The authors declare that they have no known competing financial interests or personal relationships that could have appeared to influence the work reported in this paper.

## References

[1] Pérez‐Panero AJ , Ruiz‐Muñoz M , Cuesta‐Vargas AI , Gónzalez‐Sánchez M . Prevention, assessment, diagnosis and management of diabetic foot based on clinical practice guidelines: a systematic review. Medicine. 2019;98:e16877. 31464916 10.1097/MD.0000000000016877 PMC6736276

[2] Ma RCW . Epidemiology of diabetes and diabetic complications in China. Diabetologia. 2018;61:1249‐1260. 29392352 10.1007/s00125-018-4557-7

[3] Joret MO , Dean A , Cao C , Stewart J , Bhamidipaty V . The financial burden of surgical and endovascular treatment of diabetic foot wounds. J Vasc Surg. 2016;64:648‐655. 27565588 10.1016/j.jvs.2016.03.421

[4] Armstrong DG , Swerdlow MA , Armstrong AA , Conte MS , Padula WV , Bus SA . Five year mortality and direct costs of care for people with diabetic foot complications are comparable to cancer. J Foot Ankle Res. 2020;13:16. 32209136 10.1186/s13047-020-00383-2 PMC7092527

[5] Matoori S , Veves A , Mooney DJ . Advanced bandages for diabetic wound healing. Sci Transl Med. 2021;13:eabe4839. 33731435 10.1126/scitranslmed.abe4839

[6] Glover K , Stratakos AC , Varadi A , Lamprou DA . 3D scaffolds in the treatment of diabetic foot ulcers: new trends vs conventional approaches. Int J Pharm. 2021;599:120423. 33647412 10.1016/j.ijpharm.2021.120423

[7] Cai Y , Chen K , Liu C , Qu X . Harnessing strategies for enhancing diabetic wound healing from the perspective of spatial inflammation patterns. Bioact Mater. 2023;28:243‐254. 37292231 10.1016/j.bioactmat.2023.04.019 PMC10245071

[8] Hu Y , Li H , Lv X , et al. Stimuli‐responsive therapeutic systems for the treatment of diabetic infected wounds. Nanoscale. 2022;14:12967‐12983. 36065785 10.1039/d2nr03756d

[9] Luze H , Nischwitz SP , Smolle C , Zrim R , Kamolz L‐P . The use of acellular fish skin grafts in burn wound management—a systematic review. Medicina. 2022;58:912. 35888631 10.3390/medicina58070912 PMC9323726

[10] Chen AC‐Y , Lu Y , Hsieh C‐Y , Chen Y‐S , Chang K‐C , Chang D‐H . Advanced biomaterials and topical medications for treating diabetic foot ulcers: a systematic review and network meta‐analysis. Adv Wound Care. 2023;2023:24. 10.1089/wound.2023.0024 37395488

[11] Kianian S , Zhao K , Kaur J , et al. Autologous skin grafts, versus tissue‐engineered skin constructs: a systematic review and meta‐analysis. Plast Reconstr Surg Glob Open. 2023;11:e5100. 37388427 10.1097/GOX.0000000000005100 PMC10303215

[12] Armstrong DG , Orgill DP , Galiano R , et al. A multicentre, randomised controlled clinical trial evaluating the effects of a novel autologous, heterogeneous skin construct in the treatment of Wagner one diabetic foot ulcers: interim analysis. Int Wound J. 2022;19:64‐75. 33942506 10.1111/iwj.13598 PMC8684853

[13] Ibrahim M , Ayyoubi HS , Alkhairi LA , Tabbaa H , Elkins I , Narvel R . Fish skin grafts versus alternative wound dressings in wound care: a systematic review of the literature. Cureus. 2023;15:e36348. 37082504 10.7759/cureus.36348 PMC10111873

[14] Baltzis D , Eleftheriadou I , Veves A . Pathogenesis and treatment of impaired wound healing in diabetes mellitus: new insights. Adv Ther. 2014;31:817‐836. 25069580 10.1007/s12325-014-0140-x

[15] Powers JG , Higham C , Broussard K , Phillips TJ . Wound healing and treating wounds: chronic wound care and management. J Am Acad Dermatol. 2016;74:607‐625. 26979353 10.1016/j.jaad.2015.08.070

[16] Woodrow T , Chant T , Chant H . Treatment of diabetic foot wounds with acellular fish skin graft rich in omega‐3: a prospective evaluation. J Wound Care. 2019;28:76‐80. 30767649 10.12968/jowc.2019.28.2.76

[17] Walsh JW , Hoffstad OJ , Sullivan MO , Margolis DJ . Association of diabetic foot ulcer and death in a population‐based cohort from the United Kingdom. Diabet Med. 2016;33:1493‐1498. 26666583 10.1111/dme.13054

[18] Fiakos G , Kuang Z , Lo E . Improved skin regeneration with acellular fish skin grafts. Eng Regen. 2020;1:95‐101.

[19] Coraça‐Huber DC , Steixner S , Wurm A , Nogler M . Antibacterial and anti‐biofilm activity of omega‐3 polyunsaturated fatty acids against periprosthetic joint infections‐isolated multi‐drug resistant strains. Biomedicine. 2021;9:334. 10.3390/biomedicines9040334 PMC8065983 33810261

[20] Spiegel C , Steixner SJM , Coraça‐Huber DC . Antibiofilm activity of omega‐3 fatty acids and its influence on the expression of biofilm formation genes on *Staphylococcus aureus* . Antibiotics. 2022;11:932. 35884185 10.3390/antibiotics11070932 PMC9311851

[21] Soleimani Z , Hashemdokht F , Bahmani F , Taghizadeh M , Memarzadeh MR , Asemi Z . Clinical and metabolic response to flaxseed oil omega‐3 fatty acids supplementation in patients with diabetic foot ulcer: a randomized, double‐blind, placebo‐controlled trial. J Diabetes Complications. 2017;31:1394‐1400. 28716357 10.1016/j.jdiacomp.2017.06.010

[22] McDaniel JC , Belury M , Ahijevych K , Blakely W . Omega‐3 fatty acids effect on wound healing. Wound Repair Regen. 2008;16:337‐345. 18471252 10.1111/j.1524-475X.2008.00388.x PMC2967211

[23] Magnusson S , Baldursson BT , Kjartansson H , Rolfsson O , Sigurjonsson GF . Regenerative and antibacterial properties of acellular fish skin grafts and human amnion/chorion membrane: implications for tissue preservation in combat casualty care. Mil Med. 2017;182:383‐388. 28291503 10.7205/MILMED-D-16-00142

[24] Stone R , Saathoff EC , Larson DA , et al. Accelerated wound closure of deep partial thickness burns with acellular fish skin graft. Int J Mol Sci. 2021;22:1590. 33557424 10.3390/ijms22041590 PMC7915828

[25] Kotronoulas A , Jónasdóttir HS , Sigurðardóttir RS , Halldórsson S , Haraldsson GG , Rolfsson Ó . Wound healing grafts: omega‐3 fatty acid lipid content differentiates the lipid profiles of acellular Atlantic cod skin from traditional dermal substitutes. J Tissue Eng Regen Med. 2020;14:441‐451. 31826323 10.1002/term.3005

[26] Lullove EJ , Liden B , Winters C , McEneaney P , Raphael A , Lantis Ii JC . A multicenter, blinded, randomized controlled clinical trial evaluating the effect of omega‐3‐rich fish skin in the treatment of chronic, nonresponsive diabetic foot ulcers. Wounds. 2021;33:169‐177. 33872197 10.25270/wnds/2021.169177

[27] Schaper NC , Andros G , Apelqvist J , et al. Specific guidelines for the diagnosis and treatment of peripheral arterial disease in a patient with diabetes and ulceration of the foot 2011. Diabetes Metab Res Rev. 2012;28:236‐237. 10.1002/dmrr.2252 22271745

[28] Kerr M , Rayman G , Jeffcoate WJ . Cost of diabetic foot disease to the National Health Service in England. Diabet Med. 2014;31:1498‐1504. 24984759 10.1111/dme.12545

[29] Nussbaum SR , Carter MJ , Fife CE , et al. An economic evaluation of the impact, cost, and Medicare policy implications of chronic nonhealing wounds. Value Health. 2018;21:27‐32. 29304937 10.1016/j.jval.2017.07.007

[30] Tchero H , Kangambega P , Lin L , et al. Cost of diabetic foot in France, Spain, Italy, Germany and United Kingdom: a systematic review. Ann Endocrinol. 2018;79:67‐74. 10.1016/j.ando.2017.11.005 29544659

[31] Lantis Ii JC , Lullove EJ , Liden B , et al. Final efficacy and cost analysis of a fish skin graft vs standard of care in the management of chronic diabetic foot ulcers: a prospective, multicenter, randomized controlled clinical trial. Wounds. 2023;35:71‐79. 37023475

[32] Winters C , Kirsner RS , Margolis DJ , Lantis JC . Cost effectiveness of fish skin grafts versus standard of care on wound healing of chronic diabetic foot ulcers: a retrospective comparative cohort study, wounds‐Compend. Clin Res Pract. 2020;32:283. 33370245

[33] Ontoria‐Oviedo I , Amaro‐Prellezo E , Castellano D , et al. Topical administration of a marine oil rich in pro‐resolving lipid mediators accelerates wound healing in diabetic db/db mice through angiogenesis and macrophage polarization. Int J Mol Sci. 2022;23:9918. 36077316 10.3390/ijms23179918 PMC9456080

[34] Alexander JW , Supp DM . Role of arginine and Omega‐3 fatty acids in wound healing and infection. Adv Wound Care. 2014;3:682‐690. 10.1089/wound.2013.0469 PMC4217020 25371851

[35] Vergili‐Nelsen JM . Benefits of fish oil supplementation for hemodialysis patients. J Am Diet Assoc. 2003;103:1174‐1177. 12963947 10.1016/s0002-8223(03)00984-2

[36] Burger B , Sagiorato RN , Silva JR , et al. Eicosapentaenoic acid‐rich oil supplementation activates PPAR‐γ and delays skin wound healing in type 1 diabetic mice. Front Immunol. 2023;14:1141731. 37359536 10.3389/fimmu.2023.1141731 PMC10289002

[37] Di Mitri M , Di Carmine A , Thomas E , et al. Fish skin graft: narrative review and first application for abdominal wall dehiscence in children. Plast Reconstr Surg Glob Open. 2023;11:e5244. 37718992 10.1097/GOX.0000000000005244 PMC10501472

[38] Song X , Li Z , Li Y , Hou H . Typical structure, biocompatibility, and cell proliferation bioactivity of collagen from tilapia and Pacific cod. Colloids Surf B Biointerfaces. 2022;210:112238. 34838415 10.1016/j.colsurfb.2021.112238

[39] Ramakrishnan SR , Jeong C‐R , Park J‐W , Cho S‐S , Kim S‐J . A review on the processing of functional proteins or peptides derived from fish by‐products and their industrial applications. Heliyon. 2023;9:e14188. 36938382 10.1016/j.heliyon.2023.e14188 PMC10015205

[40] Li J , Wang M , Qiao Y , et al. Extraction and characterization of type I collagen from skin of tilapia (*Oreochromis niloticus*) and its potential application in biomedical scaffold material for tissue engineering. Process Biochem. 2018;74:156‐163.

[41] Costa FJP , Nave M , Lima‐Sousa R , et al. Development of thiol‐maleimide hydrogels incorporating graphene‐based nanomaterials for cancer chemo‐photothermal therapy. Int J Pharm. 2023;635:122713. 36764414 10.1016/j.ijpharm.2023.122713

[42] Dias MTPM , Bilhar APM , Rios LC , et al. Neovaginoplasty using Nile tilapia fish skin as a new biologic graft in patients with Mayer‐Rokitansky‐Küster‐Hauser syndrome. J Minim Invasive Gynecol. 2020;27:966‐972. 31546063 10.1016/j.jmig.2019.09.779

[43] Dias MTPM , Bilhar APM , Rios LC , et al. Neovaginoplasty for radiation‐induced vaginal stenosis using Nile tilapia fish skin as a biological graft. J Surg Case Rep. 2019;2019:rjz311. 31768241 10.1093/jscr/rjz311 PMC6865336

[44] Lv K , Wang L , He X , Li W , Han L , Qin S . Application of tilapia skin acellular dermal matrix to induce acute skin wound repair in rats. Front Bioeng Biotechnol. 2022;9:792344. 35237588 10.3389/fbioe.2021.792344 PMC8882825

[45] Elshahawy AM , Mahmoud GA‐E , Mokhtar DM , Ibrahim A . The optimal concentration of silver nanoparticles in sterilizing fish skin grafts. Sci Rep. 2022;12:19483. 36376399 10.1038/s41598-022-23853-y PMC9663429

[46] López‐Nájera D , Rubio‐Zaragoza M , Sopena‐Juncosa JJ , et al. Effects of plasma rich in growth factors (PRGF) on biomechanical properties of *Achilles tendon* repair. Knee Surg Sports Traumatol Arthrosc. 2016;24:3997‐4004. 26272059 10.1007/s00167-015-3725-2

[47] Torul D , Bereket MC , Onger ME , Altun G . Comparison of the regenerative effects of platelet‐rich fibrin and plasma rich in growth factors on injured peripheral nerve: an experimental study. J Oral Maxillofac Surg. 1823;76(2018):e1‐1823.e12. 10.1016/j.joms.2018.04.012 29763577

[48] Anitua E , Pino A , Orive G . Plasma rich in growth factors promotes dermal fibroblast proliferation, migration and biosynthetic activity. J Wound Care. 2016;25:680‐687. 27827279 10.12968/jowc.2016.25.11.680

[49] Biazar E , Heidari Keshel S , Rezaei Tavirani M , Kamalvand M . Healing effect of acellular fish skin with plasma rich in growth factor on full‐thickness skin defects. Int Wound J. 2022;19:2154‐2162. 35441469 10.1111/iwj.13821 PMC9705163

[50] Abbasnezhad S , Biazar E , Aavani F , Kamalvand M , Heidari Keshel S , Pourjabbar B . Chemical modification of acellular fish skin as a promising biological scaffold by carbodiimide cross‐linker for wound healing. Int Wound J. 2023;20:1566‐1577. 36372945 10.1111/iwj.14012 PMC10088853

[51] Moura LIF , Dias AMA , Carvalho E , de Sousa HC . Recent advances on the development of wound dressings for diabetic foot ulcer treatment‐a review. Acta Biomater. 2013;9:7093‐7114. 23542233 10.1016/j.actbio.2013.03.033

[52] Wong CH , Siah KW , Lo AW . Estimation of clinical trial success rates and related parameters. Biostatistics. 2019;20:273‐286. 29394327 10.1093/biostatistics/kxx069 PMC6409418

[53] Dueppers P , Bozalka R , Kopp R , et al. The use of intact fish skin grafts in the treatment of necrotizing fasciitis of the leg: early clinical experience and literature review on indications for intact fish skin grafts. J Clin Med. 2023;12:6001. 37762941 10.3390/jcm12186001 PMC10532083

[54] Zehnder T , Blatti M . Faster than projected healing in chronic venous and diabetic foot ulcers when treated with intact fish skin grafts compared to expected healing times for standard of care: an outcome‐based model from a Swiss hospital. Int J Low Extrem Wounds. 2022;2022:153473462210962. 10.1177/15347346221096205 35546101

[55] Moustgaard H , Clayton GL , Jones HE , et al. Impact of blinding on estimated treatment effects in randomised clinical trials: meta‐epidemiological study. Brit Med Journal. 2020;368:l6802. 31964641 10.1136/bmj.l6802 PMC7190062

[56] Mathur A , Jarrett P , Broadbent E , Petrie KJ . Open‐label placebos for wound healing: a randomized controlled trial. Ann Behav Med. 2018;52:902‐908. 30212845 10.1093/abm/kax057

[57] Vits S , Dissemond J , Schadendorf D , et al. Expectation‐induced placebo responses fail to accelerate wound healing in healthy volunteers: results from a prospective controlled experimental trial. Int Wound J. 2015;12:664‐668. 24373522 10.1111/iwj.12193 PMC7950668

[58] Lau CS , Hassanbhai A , Wen F , et al. Evaluation of decellularized tilapia skin as a tissue engineering scaffold. J Tissue Eng Regen Med. 2019;13:1779‐1791. doi:10.1002/term.2928 31278852

